# Interactions Between *ABCB1* Genotype and Preoperative Statin Use Impact Clinical Outcomes Among Breast Cancer Patients

**DOI:** 10.3389/fonc.2018.00428

**Published:** 2018-10-12

**Authors:** Helga Tryggvadottir, Louise Huzell, Emma Gustbée, Maria Simonsson, Andrea Markkula, Karin Jirström, Carsten Rose, Christian Ingvar, Signe Borgquist, Helena Jernström

**Affiliations:** ^1^Clinical Sciences in Lund, Oncology and Pathology, Lund University, Lund, Sweden; ^2^Department of Hematology, Oncology, and Radiation Physics, Skåne University Hospital, Lund, Sweden; ^3^CREATE Health and Department of Immunotechnology, Lund University, Lund, Sweden; ^4^Clinical Sciences in Lund, Surgery, Lund University and Skåne University Hospital, Lund, Sweden

**Keywords:** breast cancer, statins, *ABCB1* genotype, pharmacogenetics, HMG-CoA reductase, immunohistochemistry

## Abstract

Multiple clinical trials investigate statins' effects in breast cancer. The *ABCB1* genotype appears to influence statin response and toxicity in the cardiovascular setting. This exploratory study aimed to investigate the interplay between preoperative statin use, *ABCB1* genotype, and tumor-specific expression of the statin target 3-hydroxy-3-methylglutaryl-coenzyme A reductase (HMGCR) in breast cancer. Preoperative statin use, *ABCB1* C3435T genotype, and HMGCR expression in relation to outcome were analyzed in 985 primary breast cancer patients from a population-based prospective cohort in Sweden from 2002 to 2012. Preoperative statin use (*n* = 80) was not associated with *ABCB1* C3435T genotype (*n* = 576), HMGCR expression (*n* = 848), or clinical outcomes. *ABCB1* C3435T TT-carriers had lower risk of breast cancer events than any C-carriers (adjusted hazard ratio (HR_adj_) 0.74; 95%CI 0.49, 1.12), but only in non-statin users (*P*_*interactio*__n_ = 0.042). Statin users with TT genotype had higher risk of distant metastasis (HR_adj_ 4.37; 95%CI 1.20, 15.91; *P*_*interaction*_ = 0.009) and shorter overall survival than other patients (HR_adj_ 3.77; 95%CI 1.37, 10.39; *P*_*interactio*__n_ = 0.019). In conclusion, there were nominally significant interactions between *ABCB1* genotype and preoperative statin use on clinical outcomes, while preoperative statin use was not associated with outcomes. Since this is an exploratory study of the impact of the *ABCB1* genotype in relation to statin use and clinical outcomes in the breast cancer setting, the results should be interpreted with caution and warrant replication in an independent cohort, preferably in a randomized setting. Since statin use is common in breast cancer patients, it would be of interest to further elucidate the clinical impact of the *ABCB1* genotype in breast cancer.

## Introduction

Statins are most commonly used as cholesterol-lowering agents, but there is also increasing evidence that these drugs have anti-carcinogenic effects (1, 2). Statins have well-described pleiotropic effects and have been shown to induce growth arrest or apoptosis in tumor cells and inhibit migration, inflammation and angiogenesis (2, 3). A large Danish epidemiologic study found that cancer-related mortality was reduced by up to 15% among statin users in patients with any type of cancer (4). In breast cancer, consistent evidence demonstrates that statin users have a reduced recurrence-risk (5–10). Multiple ongoing clinical trials investigate the role of statins in breast cancer (for example, NCT02483871, NCT02958852, and NCT01988571).

The benefits of statins in coronary artery disease are well-established (11). Although statin treatment is considered to be safe, side effects do occur and include myopathy, which rarely leads to severe rhabdomyolysis (12). The lipid-lowering response to statins is individual, which constitutes a problem in clinical practice (13, 14). To a large extent, the considerable inter-individual variation in drug responses has been considered to be caused by genetic factors (15). Polymorphisms in various genes have been linked to statin effectiveness and adverse effects (13, 16–20). One of the most studied genes in relation to the response to multiple drugs, including statins, is multi-drug resistance gene 1 (*MDR1* or *ABCB1*) that encodes the membrane transport protein permeability glycoprotein (P-gp). P-gp is a member of the adenosine triphosphate (ATP)-binding cassette family. P-gp is involved in the energy-dependent cellular efflux of various substrates, including drugs and various lipids (15, 21). The function of P-gp appears to be modulated by the lipid environment and its interaction with the cell membrane (22). P-gp is often overexpressed in cancer cells, including breast cancer, and this overexpression can lead to a more rapid efflux of drug out of the cells, in addition to resistance to cytotoxic treatment (23, 24).

*ABCB1* polymorphisms can alter its functional expression (21, 24). In particular, the synonymous C3435T single-nucleotide polymorphism (SNP) in exon 26 (rs1045642) has been shown to affect protein structure through altered mRNA stability, with lower mRNA levels and a consequent decrease in P-gp function associated with the T-allele (21, 25, 26). The C3435T polymorphism has been studied foremost in the context of statin use in patients with hypercholesterolemia and vascular disease, but the results have been somewhat inconsistent in terms of both effectiveness and treatment side effects, Table 1; (12, 14, 18–20, 27–33). The T-allele has been linked to an increased breast cancer risk in two small studies (34, 35) and possibly to different patient responses to chemotherapeutic agents, tamoxifen, and trastuzumab (24). While a recent large genome wide association study did not identify this SNP as an independent breast cancer risk modifier (36), another recent genome wide association study identified the candidate gene *ABCB1* as a possible effect modifier of statins on breast cancer risk in postmenopausal women via another SNP (rs9282564) near *ABCB1* (37).

**Table 1:** Selected studies investigating effects of the *ABCB1* C3435T genotype in statin users.

		**Patient population**			**Treatment**		**Outcome**	
**Author, year**	**Country**	**Population**	**No. of patients**	**Gender (female %)**	**Type of statin**	**Study period**	**Endpoints**	**Main results**
Becker et al. (2010) (27) Population-based cohort study	Netherlands	Patients prescribed statins^a^	1239		Atorvastatin or Simvastatin	16.5 years	Dose decrease Switch to another statin	Non-significant increase in risk for the variant T allele.
Ferrari et al. (2013) (12) Case-control study	Italy	Statin users	66	60.6	Atorvastatin or Rosuvastatin or Simvastatin	NA	Elevated serum CK concentration of >3*UNL	Significant trend across genotypes toward a higher risk of CK elevation in patients with T allele.
		Cases^b^	33					
		Controls^c^	33				Changes in lipid levels	T allele associated with a significantly higher reduction of LDL-C levels.
Fiegenbaum et al. (2005) (18) Clinical study	Brazil European descent	Hypercholesterolemic patients	116	75.9	Simvastatin 20 mg/day	6 months	ADR	T allele was less frequent in subjects with myalgia than in the non-ADR group (*P* < 0.05)
			99	74.7			Changes in lipid levels	T allele associated with a greater reduction of TC levels, however, not significant (*P =* 0.089).
Hoenig et al. (2011) (32) Clinical study	Australia	High-risk vascular patients	117		Atorvastatin 80 mg/day	6 weeks	Myalgia	Greater-than-expected T allele frequency in patients with myalgia compared to no myalgia.
			98	21			Changes in lipid levels	Patients with T allele showed a greater reduction in LDL-C than patients with CC (*P* = 0.034).
Kadam et al. (2016) (33) Clinical study	India	Hypercholesterolemic patients	177	28.2	Atorvastatin 10 mg/day	8 weeks	Changes in lipid levels	Patients with T allele showed a greater reduction in LDL-C (*P* < 0.05).
Kajinami et al. (2004) (14) Randomized, placebo-controlled double-blind, Atorvastatin arm	USA	Hypercholesterolemic patients	138^d^	100	Atorvastatin 10 mg/day	52 weeks	Changes in lipid levels	T allele linked to significantly lower increase of HDL-C in a gene dose-dependent manner and was associated with a larger reduction in LDL-C.
Munshi (2012) (28) Case-control study	India	Ischemic stroke patients	525	28.8	Atorvastatin 10–80 mg/day^e^	12 months	Statin response based on clinical outcome after stroke	TT genotype significantly associated with non- response to atorvastatin treatment (*P* < 0.001).
Poduri et al. (2010) (19) Clinical study	India	CAD patients	265	16.2	Atorvastatin 20 mg/day	12 months	Occurence of MI	Higher frequency of TT genotype in patients who had an MI event within a year of starting statins.
							Changes in lipid levels	No significant association between *ABCB1* C3435T genotype and changes in lipid levels.
Rodrigues et al. (2005) (29) Clinical study	Brazil European descent	Hypercholesterolemia patients	69	59.4	Atorvastatin 10 mg/day	4 weeks	Changes in lipid levels	No significant association between *ABCB1* C3435T genotype and changes in lipid levels.
								Higher baseline levels of TC and LDL-C associated with T allele, however not significant.
Rosales et al. (2012) (20) Clinical study	Chile	Hypercholesterolemia patients	142	37.3	Atorvastatin 10 mg/day	1 month	Changes in lipid levels	No significant association found between *ABCB1* C3435T genotype and reduction in lipid levels.
Salacka et al. (2014) (30) Pilot study	Poland	Patients with lipid disorder	130	67	Atorvastatin 10-20 mg/day or Simvastatin 20-40 mg/day	NA	Changes in lipid levels	Patients with CC or CT genotypes showed no significant changes in in HDL-C concentration, while patients with the TT genotype showed on average an 7 % decrease (*P* = 0.017)
Shabana et al. (2013) (31) Clinical study	Egypt	Hypercholesterolemic patients	23	100	Atorvastatin 40 mg/day	4 weeks	Changes in lipid levels	No statistically significant association found between *ABCB1* C3435T genotype and reduction in lipid levels.

a *Information obtained from pharmacy records*.

b *With statin-induced serum CK elevation*.

c *Statin users without elevated serum CK levels*.

d *Results divided by sex, only women shown here*.

e *Dose decided by neurologist*.

*ADR, adverse drug reaction; TC, Total cholesterol; LDL-C, Low-density lipoprotein cholesterol; HDL-C, High-density lipoprotein cholesterol; TG, Triglycerides; CK, Creatinin kinase; ADR, adverse drug reaction; CAD, Coronary artery disease; MI, myocardial infarction; NA, Not applicable; UNL, upper normal limit*.

The target of statins is 3-hydroxy-3-methylglutaryl-coenzyme A reductase (HMGCR), which is the rate-limiting enzyme of the mevalonate pathway (38). Prior studies have shown that HMGCR expression is often elevated or deregulated in cancer cells (39, 40). Observational data suggest that HMGCR expression may be associated with less aggressive tumor characteristics (41, 42) and a good prognosis in breast cancer patients (43). A window-of-opportunity study showed up-regulated gene expression among genes involved in apoptotic and MAPK pathways, indicating statin-induced anti-tumor effects (44). In addition, reduced tumor proliferation in HMGCR-expressing tumors prior to treatment suggested that HMGCR may be predictive of the statin response (45).We therefore hypothesized that both tumor-specific HMGCR expression and the germline *ABCB1* C3435T polymorphism may impact the effects of statins with regard to breast cancer prognosis. This exploratory study aimed to investigate the interplay between preoperative statin use, the *ABCB1* C3435T polymorphism, and tumor-specific HMGCR expression in relation to breast cancer-free and distant metastasis-free intervals and overall survival.

## Patients and methods

### Study population

**Figure 1:**
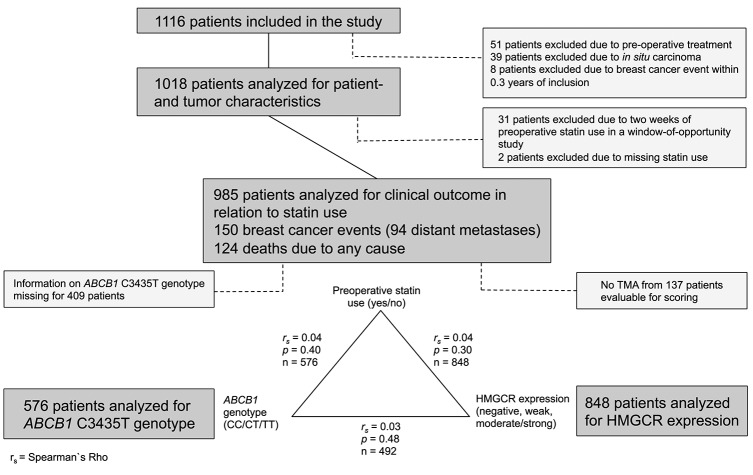
Flow chart of the number of patients included in various analyses. The number of events are indicated. The correlations between HMGCR expression, *ABCB1* genotype and preoperative statin use as well as the number of patients in each group are presented.

Women diagnosed with a primary breast cancer at Skåne University Hospital in Lund, Sweden between October 2002 and June 2012 were invited to take part in an ongoing prospective cohort study: the Breast Cancer (BC) Blood study. Patients with any previous breast cancer diagnosis or another cancer diagnosis during the previous 10 years were not eligible for participation. During the inclusion period, 1,116 patients were included in the study and followed until June 30th, 2016. Patients with preoperative treatment, *in situ* carcinoma, and early breast cancer events within 3 months of inclusion were excluded, as were patients who failed to provide information about statin use and patients who were included in a window-of-opportunity study that involved a 2-weeks preoperative statin treatment. A flow-chart of the remaining 985 study patients and the selection criteria for each analysis is presented in Figure 1.

All patients completed questionnaires preoperatively and postoperatively as described previously (41). The questionnaires included questions concerning medication intake during the past week, lifestyle, and reproductive factors. Medications were coded according to the Anatomic Therapeutic Chemical (ATC) classification system codes; code C10AA was used for statins, 92.5% were using lipophilic statins (simvastatin or atorvastatin). Smoking included preoperative current smoking and occasional smoking as described previously (46). A research nurse obtained body measurements as described previously (41).

The tumor characteristics were acquired from the patients' pathology reports. Estrogen receptor (ER) and progesterone receptor (PgR) expression were analyzed in the Department of Pathology at Skåne University Hospital in Lund, Sweden, as described previously (41). Tumors with >10% positive nuclear staining were considered to be ER-positive or PgR-positive, according to current Swedish clinical guidelines. For breast cancer patients treated at the clinic in Lund, human epidermal growth factor receptor 2 (HER2) was routinely analyzed as of November 2005 in patients younger than 70 years of age as previously described (47).

Information about treatments and breast cancer events was obtained from patient charts and the regional tumor registry. Adjuvant treatment was prescribed according to the standard of care. Breast cancer events included all types of recurrences, including ipsilateral, contralateral, regional, and distant metastases. The date of death was collected from the Swedish Population Registry.

The study was approved by the Lund University Ethics Committee (Dnr75-02, Dnr37-08, Dnr658-09, Dnr58-12, Dnr379-12, Dnr227-13, Dnr277-15, and Dnr458-15). All patients signed a written informed consent form. The study adhered to the Reporting Recommendations for Tumor Marker Prognostic Studies (REMARK) criteria (48).

### Tissue microarray construction and immunohistochemistry

Tumor tissue microarrays (TMAs) were stained with HMGCR antibody (Cat. No HPA008338, Atlas Antibodies AB, Stockholm, Sweden) (diluted 1:100) and cytoplasmic tumor-specific HMGCR protein expression was evaluated by two evaluators (EG, HT) and in cases of discrepancy a senior evaluator (SB) was consulted until consensus was reached as described previously (41). Scores were assigned based on staining intensity as follows: negative = 0, weak = 1, moderate = 2 and strong = 3. Since only 22 tumors showed a strong intensity of HMGCR expression, this group was combined with tumors that expressed HMGCR with a moderate intensity (*n* = 180). A total of 12 of the 848 included patients with available HMGCR staining had bilateral tumors. Of these patients, nine patients had evaluable cores from at least one tumor and six patients had evaluable cores from both tumors, all of which were concordant.

### Genotyping

The Wizard Genomic DNA purification kit (Promega, Madison, WI, USA) was used according to the manufacturer's protocol to obtain deoxyribonucleic acid (DNA) from buffy coats. The *ABCB1* C2435T SNP (rs1045642) was analyzed at the Region Skåne Competence Centre of Skåne University Hospital in Malmö, Sweden. The genotype analyses were performed according to the manufacturer's instructions, with the reagents included in the iPLEX^TM^ genotyping kit (Sequenom, Inc., San Diego, CA, USA) and the software and equipment in the MassARRAY® platform (Sequenom, Inc., San Diego, CA, USA). The concordance was 100% for the approximately 10% of samples that were run in duplicate. The genotypes were in Hardy Weinberg Equilibrium (chi-square = 0.36, *P* = 0.55). Genotyping was performed in 2008 and genotypes were only available for patients included in the study between October 2002 and 2008.

### Statistics

The statistical analyses were performed using SPSS Statistics 22 (IBM, Chicago, IL, USA). Patient and tumor characteristics and adjuvant breast cancer treatment prior to the last follow-up and prior to any breast cancer event were analyzed in relation to preoperative statin use and *ABCB1* C3435T genotype. The chi-square test was used for categorical variables and the linear-by-linear test for trend. The Jonkheere-Terptra and Mann-Whitney U-tests were used for continuous variables, as not all variables were normally distributed. The breast cancer-free interval was calculated from the date of inclusion until the first breast cancer event. In patients with no breast cancer events, the breast cancer-free interval was calculated using the date of the last study questionnaire or death before July 1st, 2016. Non-breast cancer-related deaths were censored at the time of death. Univariable survival analyses were calculated using Log-Rank tests. Cox proportional hazard regression was used for multivariable testing, with adjustments for age at inclusion (continuous), body mass index (BMI; continuous), tumor size (≥21 mm or skin or muscular involvement independent of size), axillary lymph node involvement (yes), histological grade III (yes), ER positivity (yes), and alcohol abstainer (yes). An interaction variable between the *ABCB1* TT genotype and statin use was created to determine if there were any effect modifications. In case of bilateral tumors, all multivariable models of HMGCR were adjusted for tumor characteristics of the corresponding side.

Power calculations based on 960 patients, of whom 80 received preoperative statin treatment with a 10 years accrual time and an additional follow-up of 4 years, revealed that true HRs ≤ 0.674 or ≥1.560 were detectable with 80% power and an alpha of 0.05. For the genotype analyses, power calculations based on 560 patients, of whom 160 had the variant genotype, and an accrual time of 6 years and an additional follow-up of 8 years after the accrual interval revealed that we will be able to detect true HRs ≤ 0.725 or ≥1.428, with 80% power and an alpha of 0.05 (49).

All statistical tests were two-tailed. *P* < 0.05 were regarded as statistically significant. Since this is an exploratory study, nominal *P*-values are presented without adjustment for multiple testing (50, 51). The first breast cancer event was considered as the primary endpoint. Distant metastasis and overall survival were considered to be secondary endpoints.

## Results

### Patient and tumor characteristics

Of the 985 patients analyzed for clinical outcome, 80 (8.1%) used statins preoperatively. HMGCR expression was available for 848 (86.1%) patients. *ABCB1* genotype data was available for 576 (58.5%) patients [TT: 172 (29.9%), CT: 292 (50.7%), CC: 112 (19.4%)]. As presented in Figure 1, statin use, HMGCR expression and *ABCB1* genotype were not correlated with each other. The patient- and tumor characteristics as well as adjuvant breast cancer treatment are presented in relation to statin use in Table 2. Preoperative statin use was positively associated with age (*P* < 0.001), BMI (*P* < 0.001), and waist-to-hip ratio (WHR) (*P* < 0.001), but not with tumor characteristics or adjuvant breast cancer treatment prior to any event or last follow-up.

**Table 2:** Patient, tumor, and treatment characteristics at inclusion in relation to preoperative statin use.

			**Preoperative statin use**	
	**All**	**Missing**	**No**	**Yes**
	***n =* 985 Median (IQR) or (%)**		***n* = 905 Median (IQR) or %**	***n* = 80 Median (IQR) or %**
Age at inclusion, years	61.0 (52.2–68.1)	0	60.3 (51.7–67.6)	67.2 (62.9–72.9)
Body mass index (BMI), kg/m^2^	25.0 (22.5–28.3)	24	24.8 (22.3–28.0)	26.7 (24.3–30.0)
Waist-to-hip ratio (WHR)	0.85 (0.80–0.90)	33	0.85 (0.80–0.90)	0.90 (0.85–0.95)
Total breast volume, mL^a^	1000 (650–1500)	154	1000 (650–1500)	1150 (700–1600)
Nulliparous	118 (12.0)	0	109 (12.0)	9 (11.3)
Current smoker	199 (20.2)	2	185 (20.4)	14 (17.5)
Alcohol abstainer	104 (10.6)	2	94 (10.4)	10 (12.5)
Ever treatment for menopausal symptoms	437 (44.5)	2	396 (43.8)	41 (51.2)
Preoperative statin use	80 (8.1)	0	0 (0)	80 (100)
Invasive tumor size		0		
1– 20 mm	722 (73.3)		667 (73.7)	55 (68.8)
21– 50 mm	248 (25.2)		224 (24.8)	24 (30.0)
>50 mm	13 (1.3)		12 (1.3)	1 (1.3)
Skin or muscular involvement	2 (0.2)		2 (0.2)	0 (0.0)
Axillary node involvement		2		
None	606 (61.6)		557 (61.7)	49 (61.3)
1–3	294 (29.9)		267 (29.6)	27 (33.8)
4+	83 (8.4)		79 (8.7)	4 (5.0)
Histological grade		1		
I	247 (25.1)		226 (25.0)	21 (26.3)
II	495 (50.3)		459 (50.8)	36 (45.0)
III	242 (24.6)		219 (24.2)	23 (28.7)
Hormone receptor status	
ER+	864 (87.8)	1	796 (88.0)	68 (86.1)
PgR+	696 (70.7)	1	644 (71.2)	52 (65.8)
HER2 amplification^b^		286		
HER2 positive	78 (11.2)		73 (11.4)	5 (8.3)
HMGCR expression		137		
Negative	111 (13.1)		103 (13.2)	8 (11.9)
Weak	535 (63.1)		496 (63.5)	39 (58.2)
Moderate/strong	202 (23.8)		182 (23.3)	20 (29.9)
Treatment by last follow–up	
Ever chemotherapy	246 (25.0)	0	232 (25.6)	14 (17.5)
Ever radiotherapy	620 (62.9)	0	568 (62.8)	52 (65.0)
Ever trastuzumab^c^	60 (76.9)	0	55 (75.0)	5 (100.0)
ER+ only		122	
Ever endocrine therapy	672 (78.0)	2	617 (77.7)	55 (80.9)
Ever tamoxifen	531 (61.6)	2	494 (62.2)	37 (54.4)
Ever aromatase inhibitor	349 (40.4)	1	320 (40.3)	29 (42.6)
Type of event	
Any breast cancer event	150 (15.2)	0	138 (15.2)	12 (15.0)
Distant metastasis	94 (9.5)	0	89 (9.8)	5 (6.3)
Death	124 (12.6)	0	111 (12.3)	13 (16.3)

a *Breast volume was not analyzed for women with previous breast surgeries*.

b *HER2 status was only available for patients younger than 70 years of age and included as of November 2005. Patients included before November 2005 were therefore missing (n = 286). HER2 status was not evaluated for additional 49 patients*.

c *Trastuzumab is presented for patients included as of November 2005 with HER2 positive tumors*.

*IQR, Interquartile range*;

*ER, Estrogen receptor; PgR, Progesterone receptor; HER2, Human epidermal growth factor receptor*.

The patient- and tumor characteristics as well as adjuvant breast cancer treatment prior to the last follow-up are presented in relation to *ABCB1* genotypes in Table 3. PgR was not evenly distributed between the three categories of *ABCB1* genotypes (*P* = 0.014), but there was no trend with increasing number of T-alleles. There were no significant trends between the number of *ABCB1* T-alleles and the patient-, tumor or treatment characteristics. The *ABCB1* TT-carriers were older than any C-carriers (*P* = 0.018).

**Table 3:** Patient, tumor, and treatment characteristics at inclusion in relation to *ABCB1* C3435T genotype.

		**Genotype^d^**				
	**All**	**Missing**	**CC**	**CT**	**TT**	**Any C**	**Missing**
	***n* = 985 Median (IQR) or (%)**		***n* = 112 Median (IQR) or %**	***n* = 292 Median (IQR) or %**	***n* = 172 Median (IQR) or %**	***n* = 404 Median (IQR) or %**	***n* = 409 Median (IQR) or %**
Age at inclusion, years	61.0 (52.2–68.1)	0	59.4 (50.8–66.3)	59.0 (50.5–65.8)	61.3 (54.2–67.5)	59.1 (50.6–66.0)	64.0 (52.5–69.4)
Body mass index (BMI), kg/m^2^	25.0 (22.5–28.3)	24	24.6 (21.8–27.9)	24.8 (22.5–28.0)	24.4 (22.3–27.9)	24.8 (22.3–28.0)	25.7 (22.6–28.6)
Waist-to-hip ratio (WHR)	0.85 (0.80–0.90)	33	0.83 (0.80–0.88)	0.84 (0.78–0.89)	0.83 (0.78–0.88)	0.84 (0.79–0.89)	0.88 (0.84–0.91)
Total breast volume, mL^a^	1000 (650–1500)	154	1000 (600–1300)	1000 (600–1450)	975 (612–1600)	1000 (600–1400)	1000 (700–1600)
Nulliparous	118 (12.0)	0	95 (15.2)	251 (14.0)	26 (15.1)	58 (14.4)	34 (8.3)
Current smoker	199 (20.2)	2	23 (20.5)	66 (22.6)	32 (18.6)	89 (22.0)	78 (19.2)
Alcohol abstainer	104 (10.6)	2	13 (11.6)	33 (11.3)	17 (9.9)	46 (11.4)	41 (10.1)
Ever treatment for menopausal symptoms	437 (44.5)	2	55 (49.1)	130 (44.7)	84 (48.8)	185 (45.9)	168 (41.2)
Preoperative statin use	80 (8.1)	0	8 (7.1)	15 (5.1)	13 (7.6)	23 (5.7)	44 (10.8)
Invasive tumor size		0	
1–20 mm	722 (73.3)		84 (75.0)	207 (70.9)	132 (76.7)	291 (72.0)	299 (73.1)
21–50 mm	248 (25.2)		26 (23.2)	81 (27.7)	37 (21.5)	107 (26.5)	104 (25.4)
>50 mm	13 (1.3)		2 (1.8)	4 (1.4)	2 (1.2)	6 (1.5)	5 (1.2)
Skin or muscular involvement	2 (0.2)		0 (0.0)	0 (0.0)	1 (0.6)	0 (0.0)	1 (0.2)
Axillary node involvement		2	
None	606 (61.6)		70 (63.1)	173 (59.5)	110 (64.0)	243 (60.4)	253 (61.9)
1–3	294 (29.9)		30 (27.0)	89 (30.6)	48 (27.9)	119 (29.6)	127 (31.1)
4+	83 (8.4)		11 (9.9)	29 (10.0)	14 (8.1)	40 (10.0)	29 (7.1)
Histological grade		1
I	247 (25.1)		26 (23.4)	90 (30.8)	42 (25.4)	116 (28.8)	89 (21.8)
II	495 (50.3)		58 (52.3)	149 (51.0)	97 (56.4)	207 (51.4)	191 (46.7)
III	242 (24.6)		27 (24.3)	53 (18.2)	33 (19.2)	80 (19.9)	129 (31.5)
Hormone receptor status							
ER+	864 (87.8)	1	91 (82.0)	261 (89.4)	151 (87.8)	352 (87.3)	361 (88.3)
PgR+	696 (70.7)	1	66 (59.5)	217 (74.3)	118 (68.6)	283 (70.2)	295 (72.1)
HER2 amplification^b^		286	
HER2 positive	78 (11.2)		8 (13.1)	14 (10.1)	10 (11.1)	22 (11.0)	46 (11.2)
HMGCR expression		137
Negative	111 (13.1)		14 (14.9)	38 (15.3)	19 (12.8)	52 (15.2)	40 (11.2)
Weak	535 (63.1)		63 (67.0)	158 (63.5)	97 (65.1)	221 (64.4)	217 (61.0)
Moderate/strong	202 (23.8)		17 (18.1)	53 (21.3)	33 (22.1)	70 (20.4)	299 (27.8)
Treatment by last follow-up
Ever chemotherapy	246 (25.0)	0	25 (22.3)	51 (17.5)	30 (17.4)	76 (18.8)	140 (34.2)
Ever radiotherapy	620 (62.9)	0	70 (62.5)	171 (58.6)	109 (63.4)	241 (59.7)	270 (66.0)
Ever trastuzumab^c^	60 (76.9)	0	2 (25.0)	10 (71.4)	6 (60.0)	12 (54.5)	42 (91.3)
ER+ only		122	
Ever endocrine therapy	672 (78.0)	2	66 (72.5)	195 (75.0)	116 (76.8)	261 (74.4)	295 (81.9)
Ever tamoxifen	531 (61.6)	2	57 (62.6)	164 (63.1)	93 (61.6)	221 (63.0)	217 (60.3)
Ever aromatase inhibitor	349 (40.4)	1	36 (39.6)	104 (34.8)	65 (43.0)	140 (39.8)	144 (40.0)
Type of event	
Any breast cancer event	150 (15.2)	0	23 (20.5)	74 (25.3)	29 (16.9)	97 (24.0)	24 (5.9)
Distant metastasis	94 (9.5)	0	14 (12.5)	48 (16.4)	16 (9.3)	62 (15.3)	16 (3.9)
Death	124 (12.6)	0	22 (19.6)	57 (19.5)	24 (14.0)	79 (19.6)	21 (5.1)

a *breast volume was not analyzed for women with previous breast surgeries*.

b *HER2 status was only available for patients younger than 70 years of age and included as of November 2005. Patients included before November 2005 were therefore missing (n = 286)*.

HER2 status was not evaluated for additional 49 patients.

c *Trastuzumab is presented for patients included as of November 2005 with HER2 positive tumors*.

d *Age, BMI and WHR have increased during the time period the cohort was compiled (2002-2012) and were lower for patients included in the genotype analysis (2002-2008)*.

*ER, Estrogen receptor; PgR, Progesterone receptor; HER2, Human epidermal growth factor receptor; IQR, Interquartile range*.

### Breast cancer events and follow-up

The patients were followed for up to 13 years, with a median follow-up of 7.0 years (interquartile range 5.0–9.1 years) for the 782 patients who were alive and still at risk. Breast cancer events occurred in 150 of the patients, 94 of whom had distant metastases. A total of 124 patients died during follow-up, of whom 71 patients had a prior recorded breast cancer event.

### Preoperative statin use and HMGCR status in relation to prognosis

Preoperative statin use was not significantly associated with the breast cancer-free interval, overall (Log-Rank *P* = 0.58) or in any of the tumor-specific HMGCR expression subgroups; no staining (Log-Rank *P* = 0.32), weak (Log-Rank *P* = 0.055) or moderate/strong expression (Log-Rank *P* = 0.79). In addition, statin use was not associated with distant metastasis-free interval (Log-Rank *P* = 0.54) or overall survival (Log-Rank *P* = 0.098).

### *ABCB1* C3435T genotype in relation to prognosis

**Figure 2:**
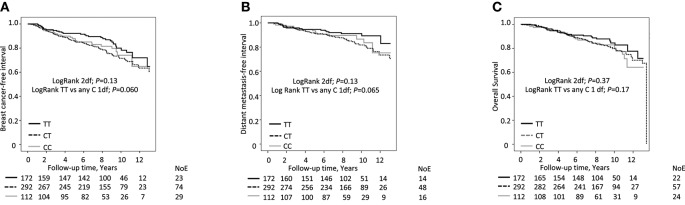
*ABCB1* C3435T genotype (TT vs. any C) in relation to **(A)** breast cancer free interval, **(B)** distant metastasis-free interval and **(C)** overall survival, respectively, according to preoperative statin use. Number of Events (NoE) and number of patients at each follow-up are indicated. Since this is an ongoing cohort, the number of patients is lower the longer the follow-up time.

No clear association was found when breast cancer-free interval was compared across the different *ABCB1* genotypes (TT, CT, and CC) (Log-Rank, 2 d.f., *P* = 0.13, Figure 2A). Since this is an exploratory data driven study and the Kaplan-Meier curves for the CC and CT groups crossed over, these groups were combined into a group of any C-carriers. TT genotype appeared inversely related to the risk of breast cancer events compared to any C-carriers but the results did not reach statistical significance and the bounds of the 95% CIs did not exclude the null (Log-Rank 1 d.f., *P* = 0.060, adjusted Hazard Ratio (HR_adj_) 0.74; 95% CI 0.49, 1.12).

Similarly, no clear association was found between the *ABCB1* genotype (TT, CT, and CC) and the distant metastasis-free interval (Log-Rank, 2 d.f., *P* = 0.13; Log-Rank TT vs. any C). Again, TT genotype appeared to be inversely related to the risk of distant metastasis compared to any C genotype but the results did not reach statistical significance [Log-Rank 1 d.f., *P* = 0.065, HR_adj_ 0.69; 95% CI 0.40, 1.21; Figure 2B]. No association between the *ABCB1* genotype and overall survival was observed, with all curves crossing each other (Log-Rank, 2 d.f., *P* = 0.37; Log-Rank TT vs. any C, 1.d.f., *P* = 0.17, Figure 2C].

### The interplay between the *ABCB1* C3435T genotype, preoperative statin use and prognosis

**Figure 3:**
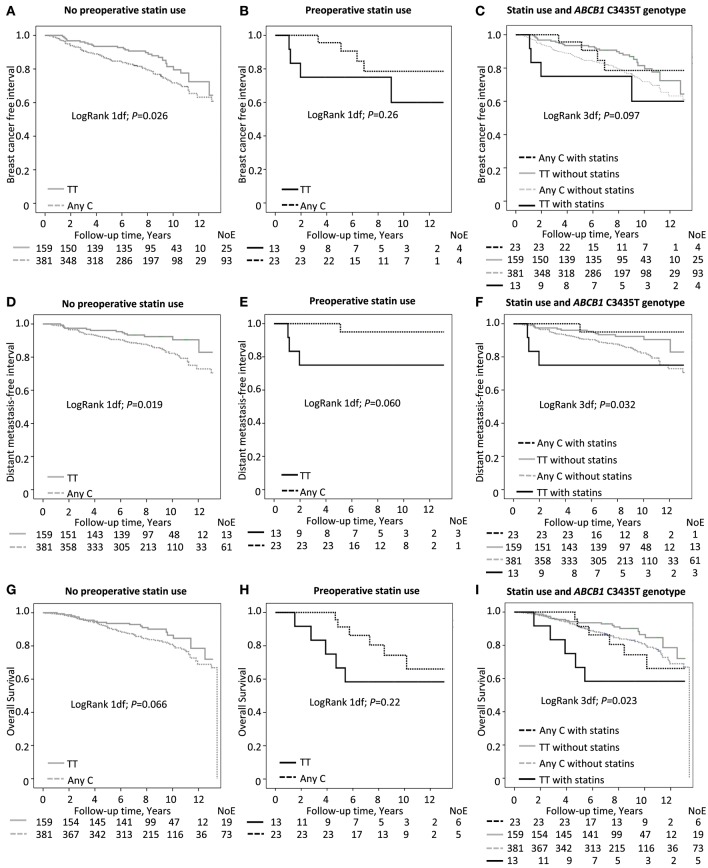
*ABCB1* C3435T genotype in relation to **(A–C)** breast cancer free interval (*P*_*interactio*__n_ = 0.042), **(D–F)** distant metastasis-free interval (*P*_*interaction*_ = 0.009) and **(G–I)** overall survival (*P*_*interactio*__n_ = 0.019), respectively. Number of Events (NoE) and number of patients at each follow-up are indicated. Since this is an ongoing cohort, the number of patients is lower the longer the follow-up time.

Since the *ABCB1* genotype may affect statin responses, a formal interaction analysis was performed to determine if there were any effect modifications of these factors on prognosis. A significant interaction between preoperative statin use and the TT genotype was found on breast cancer events (HR_adj_ 4.6; *P*_*interaction*_ = 0.042; Figure 3). In patients without preoperative statin use, the borderline association between a lower risk of breast cancer events and the TT genotype was somewhat stronger than observed for the entire group of patients in the univariable model but the bounds of the 95% CIs did not exclude the null in the multivariable model [Log-Rank *P* = 0.026; HR_adj_ 0.66 [95% CI 0.42, 1.02] Figure 3A]. In contrast, preoperative statin use in TT-carriers was not significantly associated with breast cancer events [Log-Rank *P* = 0.26; HR_adj_ 5.63 [95% CI 0.70, 44.92] Figure 3B]. When dividing the patients into four groups based on genotype and preoperative statin use, the patients with the TT genotype and preoperative statin use had an borderline increased risk of breast cancer events in comparison to the other patients in the multivariable model (Log-Rank 3 d.f. *P* = 0.097; HR_adj_ 2.98; [95% CI 1.01, 8.76] Figure 3C].

Regarding distant metastasis-free survival, in patients who had not used statins preoperatively, the *ABCB1* TT genotype was associated with a better outcome than the any C genotype [Log-Rank 1 d.f., *P* = 0.019; HR_adj_ 0.55 [95% CI 0.30, 1.00] Figure 3D]. Conversely, in preoperative statin users, the TT genotype appeared to be associated with poorer outcome but this did not reach statistical significance [Log-Rank 1 d.f., *P* = 0.060; HR_adj_ 24.90 [95% CI 0.25, 2465] Figure 3E]. As described above, when comparing the four groups of patients, with and without preoperative statin use in combination with either the TT genotype or any C genotype, the worst outcome in terms of distant metastasis was seen for preoperative statin users with the TT genotype [Log-Rank 3 d.f., *P* = 0.032; HR_adj_ 4.37 [95% CI 1.20, 15.91] Figure 3F] with a significant interaction between statin use and the TT genotype (HR_adj_ 23.9; *P*_*interaction*_ = 0.009).

The interaction between preoperative statin use and the *ABCB1* TT genotype was also significant for overall survival (HR_adj_ 5.1; *P*_*interaction*_ = 0.019). The TT genotype appeared to be positively associated with overall survival compared to any C genotype in patients without preoperative statin treatment but did not reach statistical significance [Log-Rank 1 d.f., *P* = 0.066; HR_adj_ 0.67 [95% CI 0.40, 1.13] Figure 3G]. However, overall survival was not significantly associated with the *ABCB1* genotype among preoperative statin users [Log-Rank 1 d.f., *P* = 0.22; HR_adj_ 3.02 [95% CI 0.60, 15.32] Figure 3H]. When comparing the four groups, an association was seen with overall survival; preoperative statin users with TT genotype had the worst outcomes [Log-Rank 3 d.f., *P* = 0.023; HR_adj_ 3.77 [95% CI 1.37, 10.39] Figure 3I]. Table 4 presents the multivariable interaction analysis.

**Table 4:** Multivariable analysis of preoperative statin use and *ABCB1* TT genotype and the interaction between the two variables in 576 patients.

	**Breast cancer event (*****n*** = **125 events)**			**Distant metastasis (*****n*** = **77 events)**			**Death (*****n*** = **99 events)**		
	**HR**	**95% CI**		**HR**	**95% CI**		**HR**	**95% CI**	
		**Lower**	**Upper**		**Lower**	**Upper**		**Lower**	**Upper**
TT genotype	0.66	0.42	1.02	0.55	0.30	1.01	0.68	0.40	1.13
Statin use	0.64	0.23	1.78	0.18	0.02	1.34	0.74	0.29	1.86
Interaction statin use and TT genotype	4.64	1.06	20.30	23.92	2.23	256.91	5.12	1.30	20.10
Age	0.98	0.97	1.00	1.00	0.98	1.02	1.03	1.01	1.06
BMI	1.04	1.00	1.08	1.04	0.10	1.09	1.06	1.01	1.10
Alcohol abstainer	1.47	0.87	2.47	1.57	0.83	2.95	1.36	0.76	2.44
Invasive tumor size ≥ 21 mm or muscular/skin involvement	1.74	1.18	2.56	3.16	1.96	5.12	2.26	1.49	3.42
Any axillary nodal involvement	1.20	0.82	1.74	1.63	1.01	2.64	1.49	0.98	2.27
Histological grade III	1.48	0.92	2.39	1.91	1.09	3.37	1.23	0.71	2.14
ER status	0.75	0.43	1.32	0.70	0.36	1.34	0.47	0.26	0.83

*7 patients missed one or more variables and were excluded from the multivariable analysis*.

## Discussion

In this exploratory study, patients who were *ABCB1* homozygous 3435TT-carriers and used statins preoperatively had worse outcomes than other breast cancer patients. These results suggest an unfavorable interaction between the *ABCB1* TT genotype and preoperative statin use in breast cancer patients. To the authors' knowledge, this study is the first study to investigate the interplay between preoperative statin use and the *ABCB1* C3435T genotype in breast cancer patients.

These results are partially consistent with previous studies that demonstrated that statin users who are homozygous T-allele carriers have poorer clinical outcomes in terms of cardiovascular or thrombo-embolic events (19, 28). In line with these findings, the prognosis for patients with the TT genotype appeared to differ from patients with any C genotypes in the present study, since the survival curves overlapped for patients with CC and CT genotypes. In addition, the T-allele has been linked to a higher rate of side effects from statins in some (12, 27, 32), but not all (18) studies. Moreover, this genotype has also been linked to an increased risk of breast cancer irrespective of statin use, in two small studies (34, 35). However, in a recent large genome-wide association study with over 100,000 unselected breast cancer cases this SNP and the *ABCB1* gene were not among those identified as independently relevant for breast cancer risk (36). However, in line with the results of the present study, the candidate gene *ABCB1* was identified as a possible effect modifier of statins on breast cancer risk in postmenopausal women via another SNP (rs9282564) near *ABCB1* (37).With respect to the endpoint overall survival, it would have been of interest to also study death due to cardiovascular events to get a more comprehensive picture, but this was outside the scoop of this study.

Since the *ABCB1* 3435T-allele may confer a lower mRNA expression and P-gp function (21), this genotype would be expected to lead to a reduced efflux of statins out of the cell, making statins a more effective drug in these patients. However, previous studies have reported inconsistent results. As expected, some studies showed a greater reduction of low-density lipoprotein (LDL) cholesterol and/or triglycerides in *ABCB1* 3435TT-carriers, suggesting a better response to statin treatment (12, 32). Kajinami et al. showed that a significantly larger reduction of LDL cholesterol and a smaller increase in high-density lipoprotein (HDL) cholesterol were associated with the T-allele in women but not in men, suggesting the existence of a gender-specific effect (14). On the other hand, a reduction of HDL cholesterol with statin treatment was shown in homozygous TT-carriers, suggesting a reduced treatment benefit (30). This discrepancy probably reflects the complicated interaction between P-gp and lipids, especially cholesterol, which has been shown to influence the activity and function of the drug transporter (22). By inhibiting HMGCR, statins influence intracellular cholesterol levels and circulating cholesterol levels via their hepatic actions. Statins are also substrates for active transport by membrane proteins, including the P-gp (52), indicating close interactions between statins and *ABCB1*. The *ABCB1* gene is also highly polymorphic (24, 25) and a number of other SNPs may also be of importance.

In the present study, overall preoperative statin use was not associated with breast cancer-free or distant metastasis-free intervals or overall survival. These findings stand in contrast to previous results that consistently showed a longer recurrence-free survival for breast cancer patients using statins after diagnosis (53). The vast majority of the patients in the current study used lipophilic statins that are believed to exert a larger biological effect on breast tissue than hydrophilic statins (53). Additionally, no association was found between HMGCR expression and statin use. However, recent data has revealed inconsistencies between immunohistochemistry and mRNA expression of HMGCR that can possibly be explained by lack of specificity of available antibodies against HMGCR (54). In contrast to the findings of the present study, a recent window-of-opportunity study showed an increased intensity of HMGCR expression in the majority of HMGCR-expressing tumors after a 2-weeks treatment with atorvastatin (45). The same antibody was used in the window-of-opportunity study as in the present study. Some of these patients were also included in this original cohort (41), but they were excluded from all analysis of this paper because the patients only received a 2-weeks preoperative statin treatment. Clendening and Penn previously proposed a mechanism to explain how some cancer cells may develop statin resistance (2). We speculate that patients who used statins preoperatively may have developed tumors that were less dependent on cholesterol metabolism and consequently less influenced by statin treatment. In this study, only preoperative statin use was examined and the importance of the timing of statin use in relation to breast cancer diagnosis and prognosis is still not fully understood. It is possible that some patients classified as non-users started using statins postoperatively, which would have biased the results toward the null. In contrast, concerns regarding the potential effect of selection and immortal-time bias in observational studies has been raised for studies showing survival benefits in statin users (55).

Preoperative statin use in homozygous T-allele carriers was associated with an increased risk of distant metastasis in addition to death due to any cause. Approximately half of the patients with the TT genotype and preoperative statin use who died did not have a reported breast cancer event prior to death. In contrast, among patients without preoperative statin use, homozygous T-allele carriers had a lower risk of breast cancer events than any C-carriers, and the interactions between genotype and statin use were significant. Preoperative statin use was not significantly associated with outcome in patients treated with tamoxifen, aromatase inhibitors, radiotherapy, or chemotherapy when the *ABCB1* C3435T genotype was not taken into consideration (data not shown). Although the power was decent for the whole cohort, it was smaller in the subgroup analysis and the number of preoperative statin users with the TT genotype was too small for meaningful stratification according to treatment.

This study explored the hypothesis that the *ABCB1* C3435T polymorphism confers effect modifications of statin use on three different breast cancer outcomes. Nominal two-sided *P*-values without adjustment for multiple testing are presented. Each *P*-value should therefore be viewed as the level of evidence against each null hypothesis. Since three main interactions were tested, it could be argued that adjustment for multiple testing should have been carried out, in which case only the *P*-value for interaction with regards to distant metastasis would hold. However, some statisticians have argued that data of exploratory studies should be analyzed without adjustments for multiplicity since appropriate multiple test adjustment is difficult or even impossible (50). After stratification by preoperative statin use further tests were performed to explore whether the genotype impacted on outcome in each treatment group. Several *P*-values were close to the 0.05 limit and would not hold for adjustment for multiple testing. The results must therefore be viewed with caution and confirmatory studies are warranted to elucidate whether there is a true effect modification by the *ABCB1* C3435T genotype on statin in relation to breast cancer outcomes.

An important strength of the present study is that it is population-based, which makes the results generalizable for women treated for primary breast cancer in Southern Sweden (46). In Sweden statins are not sold over the counter. Medication use was self-reported, and information on statin use was missing for only two patients. If prescription data had been used then there would be a risk of inclusion of non-adherent patients into the statin group. However, some study limitations must be noted. A limited number of participants used statins preoperatively; subsequently, a relatively small number of events occurred in the different genotype groups of statin users. Additionally, information about the participants' indications for statin use and cholesterol levels was not available.

In conclusion, this exploratory study is the first to show effect modifications between the *ABCB1* C3435T genotype and preoperative statin use on breast cancer outcomes, including breast cancer-free and distant metastasis-free intervals, and most importantly, overall survival. Preoperative statin use was not independently associated with outcome. Since no adjustments for multiple testing were performed in this exploratory study, the results need to be interpreted with caution as they may be due to chance. The findings warrant confirmation in an independent cohort and since statin use is common in breast cancer patients it would be of interest to study the impact of the *ABCB1* genotype in relation to statin use and clinical outcome in a randomized setting, to elucidate the clinical impact of the *ABCB1* genotype in breast cancer.

## Ethics statement

This study was carried out in accordance with the recommendations of the ethics committee at Lund University. All subjects gave written informed consent in accordance with the Declaration of Helsinki. The study was approved by the ethics committee at Lund University (Dnr 75-02, Dnr 37-08, Dnr 658-09, Dnr 58-12, Dnr 379-12, Dnr 227-13, Dnr 277-15, and Dnr 458-15).

## Author contributions

HT, SB, CI, and HJ: Conception and design. HJ: Development of methodology. HT, LH, EG, SB, KJ, CI, CR, and HJ: Acquisition of data. HT, MS, SB, and HJ: Analyses and interpretation of data. HT, LH, EG, MS, AM, SB, KJ, CR, CI, and HJ: Writing review/and or revision of manuscript. HT, LH, EG, MS, AM, KJ, and HJ: Administrative, technical or material support. CI, SB, and HJ: Study supervision.

### Conflict of interest statement

The authors declare that the research was conducted in the absence of any commercial or financial relationships that could be construed as a potential conflict of interest.
